# Effect of an intervention targeting inappropriate continued empirical parenteral vancomycin use: a quasi-experimental study in a region of high MRSA prevalence

**DOI:** 10.1186/s12879-018-3081-1

**Published:** 2018-04-16

**Authors:** Pyoeng Gyun Choe, Hei Lim Koo, Doran Yoon, Ji Yun Bae, Eunyoung Lee, Joo-Hee Hwang, Kyoung-Ho Song, Wan Beom Park, Ji Hwan Bang, Eu Suk Kim, Hong Bin Kim, Sang Won Park, Myoung-don Oh, Nam Joong Kim

**Affiliations:** 1 Department of Internal Medicine, Seoul National University College of Medicine, 103 Daehak-ro Jongno-gu, Seoul, 03080 Republic of Korea; 2 Infection Control Office, Seoul National University Hospital, Seoul, Republic of Korea; 3 Present address: Department of Internal Medicine, Chonbuk National University Medical School, Jeonju, Republic of Korea

**Keywords:** Vancomycin, Inappropriate use, Antimicrobial stewardship, Intervention, Pharmacist, Infectious disease specialist

## Abstract

**Background:**

Despite vancomycin use is a major risk factor for the emergence of vancomycin resistance, it is frequently inappropriately prescribed, especially as empirical treatment. We evaluated the effect of an antimicrobial stewardship intervention targeting for inappropriate continued empirical vancomycin use.

**Methods:**

This was a quasi-experimental study comparing vancomycin use in a 6-month pre-intervention and 6-month intervention period. If empirical vancomycin was continued for more than 96 h without documentation of beta-lactam-resistant gram-positive microorganisms, it was considered inappropriate continued empirical vancomycin use. The intervention consisted of the monitoring of appropriateness by a pharmacist and direct discussion with the prescribing physicians by infectious disease specialists when empirical vancomycin was continued inappropriately. An interrupted time series analysis was used to compare vancomycin use before and during the intervention.

**Results:**

Following implementation of the intervention, overall vancomycin consumption decreased by 14.6%, from 37.6 defined daily doses (DDDs)/1000 patient-days in the pre-intervention period to 32.1 DDDs/1000 patient-days in the intervention period (*P* <  0.001). The inappropriate consumption of vancomycin also declined from 8.0 DDDs/1000 patient-days to 5.8 DDDs/1000 patient-days (*P* = 0.009).

**Conclusion:**

Interventions such as direct communication with prescribing physicians and infectious disease clinicians can help reduce the inappropriate continued use of vancomycin.

## Background

Vancomycin use is a major risk factor for acquisition of vancomycin-resistant Enterococci (VRE) [1], and infectious diseases caused by VRE are associated with increased mortality and length of hospital stay [2]. Also, the use of unnecessary vancomycin exposes patients to the risk of nephrotoxicity [3]. In 1995 the Centers for Disease Control and Prevention (CDC) established guideline for the appropriate use of vancomycin to reduce vancomycin resistance [4]. Since that time, many clinical guidelines recommended that empirically initiated vancomycin should be stopped, even in immunocompromised hosts, such as those with neutropenia, if there is no evidence of beta-lactam-resistant gram-positive infection [5, 6]. Despite these guidelines, 20% to 70% of vancomycin use is inappropriate [7–11].

In our institution, over the last 12 years, about 60% of *S. aureus* infections were methicillin-resistant (MRSA), and this proportion has not changed significantly [12], whereas vancomycin consumption has increased more than twofold, from 18 defined daily doses (DDDs) per 1000 patient-days to 40.0 DDDs per 1000 patient-days [13]. Our previous analysis showed that a quarter of total vancomycin use represented inappropriately continued empirical use [14]. We have been executing an antibiotics stewardship program including an educational program, a computer-assisted antibiotic prescribing program, antimicrobial formulary restriction, and a prior-approval program. Our prior approval program is not very restrictive: physicians who are going to prescribe vancomycin have to describe the indication on a vancomycin order form which is modified from the indications for vancomycin use developed by the CDC [4]. Prescribing physicians are recommended, but not obliged to obtain prior approval by an infectious diseases specialist. In March 2015, the Antimicrobial Control Team of Seoul National University Hospital established an intervention to improve the appropriateness of empirical vancomycin use. The intervention consisted of monitoring of appropriateness by a pharmacist and direct discussion between the prescribing physicians and infectious disease specialists when empirical vancomycin was continued inappropriately beyond 96 h. We report the details of the intervention program and its effects on empirical vancomycin use.

## Methods

### Study design

This quasi-experimental study was performed in Seoul National University Hospital, a 1778-bed, university-affiliated tertiary hospital in South Korea. The study was divided into two 6-month periods with respect to the intervention targeting inappropriate continued empirical vancomycin use, which began on March 16th 2015. We defined a 6-month pre-intervention period from July 14th 2014 to January 13th 2015, with the subsequent 2-month regarded as a washout period. The intervention period was then defined as the 6-month from the start of the intervention on March 16th 2015 to September 15th 2015. The institutional review board of Seoul National University Hospital reviewed the study protocol and provided study approval. It waived the requirement for written consent (IRB registration number 1407–043-593).

### The intervention

During the intervention period, the pharmacist of the Antibiotic Control Team reviewed the medical records of patients for whom parenteral vancomycin was prescribed every day. If empirical vancomycin was continued beyond 96 h without documentation of beta-lactam-resistant gram-positive microorganisms, the pharmacist informed an infectious disease specialist of the Antibiotic Control Team that empirical vancomycin was being prescribed inappropriately. The infectious disease clinician then met with, or phoned, the prescribing physician, and urged him/her to discontinue the vancomycin. Direct communication with the relevant physicians was intended to take place within 48 h of the time when infectious disease clinician was informed of the inappropriate continued empirical vancomycin use. The infectious disease clinician left a medical record of the recommendation to the prescribing physician and the pharmacist of the Antibiotic Control Team later confirmed that vancomycin had been discontinued.

### Data collection and definitions

We retrospectively reviewed the medical records of patients who had been prescribed at least one dose of parenterally administered vancomycin during the study period. We included only patients who were at least 18 years of age.

The primary outcomes of the study were the total amount of vancomycin prescribed, the amount of inappropriate continued empirical vancomycin use, and the total amount of vancomycin prescribed that constituted inappropriate continued empirical use. Inappropriate continued empirical vancomycin was defined as in our previous report [14]. Briefly, we considered vancomycin prescribed before obtaining the culture results to be empirical. When empirically prescribed vancomycin treatment was continued beyond 96 h without documentation of beta-lactam-resistant gram-positive microorganisms in clinical specimens with significance, the continuation was considered inappropriate, and the amount used thereafter was considered inappropriately used. Vancomycin was calculated as defined daily doses (DDDs) per 1000 patient-days in accordance with World Health Organization recommendations [15]. Prescriptions for the same patient that were separated by 8 days or more were considered independent.

### Statistical analysis

Descriptive results for continuous variables were expressed as median values and interquartile ranges (IQRs). Clinical characteristics were compared using the chi-square test and the Matt-Whitney test for categorical and continuous variables, respectively. An interrupted time series analysis was used to compare the amount of vancomycin use before and after the intervention. Data analyses were performed using SAS software (version 9.4; SAS Institute, Cary, North Carolina).

## Results

### Vancomycin consumption in the pre-intervention period

During the pre-intervention period, a total of 1450 prescriptions of parenterally administered vancomycin were provided for 1249 patients, corresponding to 37.6 DDDs/1000 patient-days. Of the 1450 prescriptions, 212 (14.6%) were for specific treatment of documented infections, 347 (23.9%) were prophylactic, and 891 (61.5%) were empirical. The amounts consumed for specific treatment, prophylaxis, and empirical treatment were 8.5 DDDs/1000 patient-days (22.5%), 5.2 DDDs/1000 patient-days (13.8%), and 23.9 DDDs/1000 patient-days (63.7%) respectively.

### Intervention activity

During the intervention period, a total of 1457 prescriptions of parenterally administered vancomycin were given to 1244 patients. Of the 1457 prescriptions, 908 (62.3%) were given empirically and 272 (18.7%) were continued inappropriately beyond 96 h and became candidates for the intervention. An infectious disease specialist intervened about 223 (82.0%) of these 272 prescriptions; 148 prescriptions (66.4%, of 223 prescriptions) were discontinued within 24 h of the intervention, but 75 (33.6%) were continued.

### Vancomycin consumption in the intervention period

**Fig. 1:**
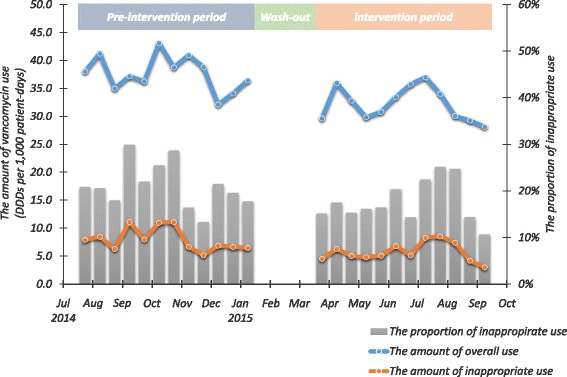
Vancomycin consumption and appropriateness in the pre-intervention and intervention periods

During the intervention period, overall vancomycin consumption decreased by 14.6%, from 37.6 DDDs/1000 patient-days in the pre-intervention period to 32.1 DDDs/1000 patient-days in the intervention period (*P* <  0.001, Fig. 1). In the latter period the amounts consumed for specific treatment, prophylaxis, and empirical treatment were 6.5 DDDs/1000 patient-days (20.2%), 5.7 DDDs/1000 patient-days (17.8%), and 19.9 DDDs/1000 patient-days (62.0%) respectively. During the intervention period, the consumption for empirical use also was declined significantly (*P* = 0.005, Table 1). The incidence of MRSA bloodstream infection was 0.112 per 1000 patient-days in the pre-intervention period and 0.147 per 1000 patient-days in the intervention period, with no significant difference between the two periods (*P* = 0.272).

**Table 1:** The usage of vancomycin in the pre-intervention and intervention periods

	Pre-intervention period	Intervention period	*P* value
Total amount of vancomycin prescribed (DDDs/1000 patient-days)	37.6	32.1	< 0.001
Amount of empirical vancomycin (DDDs/1000 patient-days)	23.9	19.9	0.005
Amount of inappropriate continued empirical vancomycin (DDDs/1000 patient-days)	8.0	5.8	0.009

*Abbreviations: DDD* defined daily dose

### Inappropriate continued empirical vancomycin use

We identified 575 patients for whom empirical vancomycin was continued inappropriately during this study: 303 in the pre-intervention period and 272 in the intervention period. The proportion of patients with hematologic malignancies among the patients in whom empirical vancomycin was continued inappropriately was significantly higher during the intervention period, but the other baseline and clinical characteristics of the two sets of patients did not differ significantly (Table 2). In the pre-intervention period, the amount of inappropriate continued empirical vancomycin use was 8.0 DDDs/1000 patient-days, representing 21.2% of the total parenterally administered vancomycin. In the intervention period, the inappropriate consumption of vancomycin declined to 5.8 DDDs/1000 patient-days (*P* = 0.009, Fig. 1). The proportion of vancomycin consumption amount that was continued inappropriately also decreased, but not significantly (21.2% to 18.1%, *P* = 0.087).

**Table 2:** Characteristics of patients in whom empirical vancomycin was continued inappropriately during the pre-intervention and intervention periods^a^

	Pre-intervention period	Intervention period	*P* value
No. of prescriptions	303	272	
Male, *n* (%)	181 (59.7)	163 (59.9)	0.963
Age, median years (IQR)	60 (48–71)	61 (49–72)	0.528
Comorbid condition, *n* (%)			
Diabetes mellitus	68 (22.4)	53 (19.5)	0.385
Chronic liver disease	28 (9.2)	27 (9.9)	0.780
Chronic lung disease	10 (3.3)	1 (0.4)	0.010
Cerebrovascular disease	10 (3.3)	11 (4.0)	0.635
Solid malignancy	86 (28.4)	68 (25.0)	0.360
Hematological malignancy	58 (19.1)	72 (26.5)	0.036
Connective tissue disease	8 (2.6)	8 (2.9)	0.827
Azotemia	46 (15.2)	46 (16.9)	0.572
Neutropenia	61 (20.1)	63 (23.2)	0.378
Suspected site of infection, *n* (%)			0.214
Pneumonia	68 (22.4)	50 (18.4)	
Intraabdominal infection	32 (10.6)	42 (15.4)	
CNS infection	34 (11.2)	32 (11.8)	
Skin and soft tissue infection	62 (20.5)	50 (18.4)	
Cardiovascular infection	2 (0.7)	5 (1.8)	
Catheter-related infection	25 (8.3)	16 (5.9)	
Bone and joint infection	31 (10.2)	8 (2.9)	
Urinary tract infection	5 (1.7)	3 (1.1)	
Other infection	19 (6.3)	19 (7.0)	
Unknown	25 (8.3)	47 (17.3)	
Admission department, *n* (%)			0.303
Medial ward	165 (54.5)	176 (64.7)	
Surgical ward	86 (28.4)	46 (16.9)	
Medical ICU	33 (10.9)	30 (11.0)	
Surgical ICU	19 (6.3)	20 (7.4)	
30-days mortality, * n* (%)	53 (17.5)	37 (13.6)	0.200

*Abbreviations: CNS* central nervous system*, ICU* intensive care unit

^a^When empirically prescribed vancomycin treatment was continued beyond 96 h without documentation of beta-lactam-resistant gram-positive microorganisms in clinical specimens with significance, the continuation was considered inappropriate

## Discussion

We have shown above that an intervention involving direct discussion between prescribing physicians and infectious disease clinicians increased the appropriateness of empirical vancomycin use. The intervention reduced the amount of inappropriate continued empirical vancomycin use from 8.0 to 5.8 DDDs/1000 patient-days, and the total amount of vancomycin prescribed represented by inappropriate continued empirical vancomycin use fell from 21.2% to 18.1%.

There have been several investigations of the effects of various interventions aimed at improving vancomycin use, including pharmacists’ interventions, automatic stop orders, antibiotic order forms, continuing education, computer-assisted antibiotic prescribing programs, antimicrobial formulary restrictions, and prior-approval programs. The effectiveness of pharmacists’ interventions was demonstrated in a previous study [16]; the initial interventions were performed by pharmacists, and if inappropriate vancomycin use continued, a consultation with an infectious disease clinician was offered. As a result, the accord with guidelines for empiric use of vancomycin improved from 47% in the pre-intervention period to 73% during the intervention [16]. Another pharmacists’ intervention consisting of contacting physicians and informing them of inappropriate vancomycin use significantly improved appropriate initiation of vancomycin [17]. These studies demonstrated that direct interventions of pharmacist and infectious disease clinicians with prescribers could be effective. An intervention consisting automatic 72 h stop orders also improved vancomycin prescribing [18]. However Bolon et al. reported that an antibiotic order form intervention did not improve or reduce vancomycin use [19], and the educational programs about vancomycin use failed to reduce inappropriate vancomycin prescribing in a prospective study [20].

An educational program, a computer-assisted antibiotic prescribing program, and a prior approval program have all been introduced in our institution to promote the prudent use of broad-spectrum antibiotics including vancomycin. Nevertheless, much of the vancomycin prescribed has been used inappropriately. In our opinion education and antibiotic order forms alone have little impact on the appropriateness of antibiotic use, and more intensive interventions such as direct discussions and compulsory stop orders would be more helpful for antimicrobial stewardship. Of course, such direct discussions with prescribing physician may not be long lasting, as it increases the demands on the time of infectious disease clinicians. Therefore, with this interventions, it is essential to establish local guidelines and policies for empirical prescribing. In Korea, to promote appropriate use of antibiotics, the Korea Centers for Disease Control and Prevention (KCDC) is carrying out a Policy Research Serving Project with related expert groups such as the Korean Society of Infectious Diseases, Korean Society for Chemotherapy since 2016 [21, 22].

Factors associated with the inappropriate use of vancomycin, such as critical clinical conditions, absence of documented causative organism, and suspected CNS infection, were identified in previous studies [7, 14]. In this study, patients with hematologic malignancies accounted for a significantly higher proportion of the patients in whom empirical vancomycin was continued inappropriately during the intervention period than during the pre-intervention period. This suggests that physicians treating patients with hematologic malignancies tend to be less compliant with intervention instructions.

Our study has several limitations. First, we only focused on reducing the amount of inappropriate continued empirical vancomycin use. Further interventions focused on prophylaxis, specific treatments, and the initial choice of empirical vancomycin would also be helpful in reducing the total amount of vancomycin prescribed. Second, this was a non-randomized, pre/post-intervention study and the lack of random assignment weakens its significance. Third, we did not investigate why many prescribers continued with inappropriate empirical vancomycin use after discussion with infectious diseases clinicians.

## Conclusions

Interventions such as direct communication with prescribing physicians and infectious disease clinicians can help reduce the inappropriate continued use of vancomycin.

## Abbreviations

CDC: Centers for disease control and prevention
DDDs: Defined daily doses
IQRs: Interquartile ranges
MRSA: Methicillin-resistant *Staphylococcus aureus*
VRE: Vancomycin-resistant enterococci
